# DMRfinder: efficiently identifying differentially methylated regions from MethylC-seq data

**DOI:** 10.1186/s12859-017-1909-0

**Published:** 2017-11-29

**Authors:** John M. Gaspar, Ronald P. Hart

**Affiliations:** 1 Department of Pharmaceutics, Rutgers University, Piscataway, NJ USA; 2 Department of Cell Biology and Neuroscience, Rutgers University, Piscataway, NJ USA

**Keywords:** DNA methylation, Bisulfite sequencing, CpG islands, Single-linkage clustering

## Abstract

**Background:**

DNA methylation is an epigenetic modification that is studied at a single-base resolution with bisulfite treatment followed by high-throughput sequencing. After alignment of the sequence reads to a reference genome, methylation counts are analyzed to determine genomic regions that are differentially methylated between two or more biological conditions. Even though a variety of software packages is available for different aspects of the bioinformatics analysis, they often produce results that are biased or require excessive computational requirements.

**Results:**

DMRfinder is a novel computational pipeline that identifies differentially methylated regions efficiently. Following alignment, DMRfinder extracts methylation counts and performs a modified single-linkage clustering of methylation sites into genomic regions. It then compares methylation levels using beta-binomial hierarchical modeling and Wald tests. Among its innovative attributes are the analyses of novel methylation sites and methylation linkage, as well as the simultaneous statistical analysis of multiple sample groups. To demonstrate its efficiency, DMRfinder is benchmarked against other computational approaches using a large published dataset. Contrasting two replicates of the same sample yielded minimal genomic regions with DMRfinder, whereas two alternative software packages reported a substantial number of false positives. Further analyses of biological samples revealed fundamental differences between DMRfinder and another software package, despite the fact that they utilize the same underlying statistical basis. For each step, DMRfinder completed the analysis in a fraction of the time required by other software.

**Conclusions:**

Among the computational approaches for identifying differentially methylated regions from high-throughput bisulfite sequencing datasets, DMRfinder is the first that integrates all the post-alignment steps in a single package. Compared to other software, DMRfinder is extremely efficient and unbiased in this process. DMRfinder is free and open-source software, available on GitHub (github.com/jsh58/DMRfinder); it is written in Python and R, and is supported on Linux.

**Electronic supplementary material:**

The online version of this article (10.1186/s12859-017-1909-0) contains supplementary material, which is available to authorized users.

## Background

Methylation at the 5-position of cytosine bases in DNA is an epigenetic modification that affects development and gene regulation. In adult mammalian cells, methylation most commonly occurs at 5′-CG-3′ dinucleotides, often termed ‘CpG’ sites. Although CpG sites occur less frequently than expected in mammalian genomes, more than half of gene promoters contain short, clustered regions with high concentrations of these sites [1]. These ‘CpG islands’ are largely unmethylated in somatic cells, but those near genes under long-term transcriptional repression are frequently methylated. Aberrant methylation patterns have been associated with cancer and other diseases [2].

Examining genomic methylation at single-base resolution is accomplished by treating DNA with bisulfite, which deaminates only unmethylated cytosines, followed by high-throughput sequencing (MethylC-seq or BS-seq). Researchers often use a targeted approach to bisulfite sequencing, such as reduced representation bisulfite sequencing (RRBS) [3] or hybridization enrichment [4, 5], to gain greater-depth methylation data in genomic areas that are likely to be of functional significance. After alignment of the sequence reads to a reference genome by a software package such as Bismark [6], methylation counts are extracted and analyzed for differential methylation. The single-base resolution of the data allows for testing individual CpG sites, but regulated methylation targets are most commonly clustered into short regions. Therefore, a more biologically appropriate approach is to determine differentially methylated regions (DMRs) of multiple CpG sites.

Although several statistical approaches exist to identify DMRs between two sample groups, many of these have substantial limitations. Using Fisher’s exact tests on methylation counts fails to account for variation between replicates, since the counts are summed within each sample group. Simple *t*-tests of methylation levels do consider this biological variation, but they ignore the binomial nature of the methylation data [7]. A third approach is to first smooth methylation levels prior to conducting *t*-tests, as implemented in the R/Bioconductor package bsseq [8], but this may lead to artifacts in regions of sparse data, especially with targeted MethylC-seq datasets.

In this paper, we introduce a software package, DMRfinder, that identifies DMRs between two (or more) sample groups in an efficient and unbiased manner. The software is compared against other computational approaches to find DMRs using a large published dataset [9].

## Implementation

**Fig. 1:**
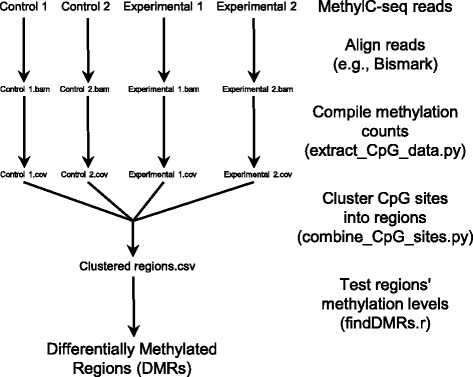
Overview of DMRfinder. A simple MethylC-seq analysis of two replicates each of control and experimental groups. After alignment of the reads, the three steps of DMRfinder ultimately produce a list of genomic regions with methylation differences between the control and experimental samples

The DMRfinder pipeline (Fig. 1) consists of three scripts, two in Python and one in R, and is run on the command-line. As input, it takes high-throughput MethylC-seq datasets that have been aligned with a software tool such as Bismark [6]. DMRfinder extracts CpG methylation counts from the alignment files, and then it clusters the CpG sites into genomic regions. The final step is to perform pairwise comparisons of the sample groups to identify DMRs. Complete descriptions of the usage and parameters of DMRfinder, along with a sample workflow, are provided in the UserGuide that accompanies the software on GitHub.

### Extracting methylation counts

The first step of DMRfinder (extract_CpG_data.py) converts the output from Bismark’s aligner (or any alternative aligner that produces a similarly formatted file) into a table of methylated/unmethylated counts at each CpG site. The output table is sorted by chromosome and position (1-based). The methylation counts are merged, in that the totals for the cytosine and the guanine of each CpG site are summed. This script of DMRfinder is not limited to canonical genomic CpG sites, so it will include counts of novel CpG sites, as indicated in the alignment file. Furthermore, this script allows for the analysis of spatially-linked methylation information in user-specified genomic intervals.

### Clustering CpG sites into regions

A modified single-linkage clustering algorithm is implemented in DMRfinder (combine_CpG_sites.py). The program determines the valid CpG sites that meet minimum coverage criteria for one or more samples. Then it groups sites that are within a specified distance of each other into regions. This single-linkage clustering can lead to the chaining effect, in which distant CpG sites are placed into the same region due an extended series of intermediate sites. The DMRfinder script limits this effect by splitting any region whose length exceeds a specified threshold (by default, 500 bp). After clustering, each region is additionally required to meet a total methylation count minimum (by default, twenty) in each sample before being tested for differential methylation (note that this parameter does not strictly correspond to read coverage, since a single read may have methylation data at multiple CpG sites). The overall clustering process is controlled exclusively by the parameters that define which CpG sites are considered valid and the distances between those sites; it does not consider methylation levels, and thus is not biased in favor of (or opposed to) finding DMRs.

### Testing regions for differential methylation

The final step of the DMRfinder pipeline (findDMRs.r) conducts pairwise tests of sample groups to identify genomic regions that are differentially methylated. The underlying statistics are based on Bayesian beta-binomial hierarchical modeling, which accounts for both the biological variation of replicates and the binomial nature of the methylation data. This is followed by Wald tests, as implemented in the R/Bioconductor package DSS [10]. Regions that meet the minimum methylation difference (by default, 10%) and the maximum *p*- and *q*-values (by default, *p* < 0.05) are reported as DMRs.

## Results and discussion

We compared the performance of DMRfinder against other software packages using the 4.8 billion reads generated by Lister et al. [9] in their study of two human cell lines, IMR90 and H1. The reads were preprocessed, aligned, and analyzed for DMRs as described in Additional file 1.

### Extracting methylation counts

The post-alignment steps of the Bismark pipeline provide comprehensive genome-wide methylation information, summarizing results based on the sequence context of every cytosine on both forward and reverse strands. For research projects focused on CpG methylation, this process can be greatly streamlined, especially when a targeted sequencing approach (such as RRBS) is employed. Hence, the first step of DMRfinder tabulates methylation counts specifically at CpG sites. To extract methylation counts from the Lister et al. [9] IMR90 replicate 1a samples, DMRfinder completed the process in less than half the time of the Bismark pipeline and required 193 times less disk space (Table 1). With datasets generated from a targeted sequencing approach, rather than genome-wide, DMRfinder is even more efficient at producing methylation counts.

**Table 1:** Comparison of production of methylation counts with Bismark and DMRfinder

	Bismark	DMRfinder
Run-time (sec)	22,514	9,378
Disk usage (Gb)	172.51	0.89

The Bismark scripts ‘bismark_methylation_extractor’ and ‘coverage2cytosine’ and the DMRfinder script ‘extract_CpG_data.py’ were used to analyze the 335.2 million aligned reads of the Lister et al. [9] IMR90 replicate 1a samples

Another important difference between DMRfinder and Bismark concerns novel CpG sites. The Bismark script ‘coverage2cytosine’ analyzes only CpG sites that are found in the reference genome. Since the canonical reference genomic sequence is not perfectly accurate for any individual, let alone for a subset of cells from a particular individual [11], determining the methylation status of known genomic CpG dinucleotides is inherently limited. Natural variants can lead to the creation of novel CpG sites, and, unlike Bismark, DMRfinder incorporates these sites into its output.

**Fig. 2:**
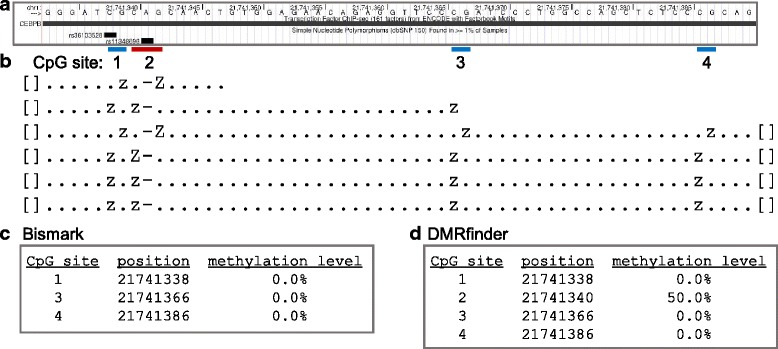
DMRfinder analyzes novel CpG sites. **a** A segment of chromosome 1 in a CEBPB binding site, as shown with the UCSC genome browser [12]. The canonical sequence has three CpG sites (blue boxes, #1, 3, and 4). The deletion of the adenine at position 21,741,341, a known variant (rs11348696) results in the creation of a novel CpG site (red box, #2). **b** The alignment of the methylation strings of six reads from the IMR90 replicate 1 dataset with the genomic segment in part A. The methylation strings show only CpG methylation status (‘z’ = unmethylated, ‘Z’ = methylated). All six reads have a deletion of the adenine at position 21,741,341 (‘-’), and thus indicate that the methylation status occurs in a CpG context. **c** With Bismark, the output summarizing methylation counts lists data for sites #1, 3, and 4, but omits the new CpG site. **d** With DMRfinder, the output includes the novel CpG site, revealing a higher methylation level in this genomic region

For example, all the reads of the IMR90 datasets that aligned to a particular genomic segment of chromosome 1 (Fig. 2a) showed a deletion of the adenine at position 21,741,341, a known variant (rs11348696, dbSNP build 150) that results in the creation of a novel CpG site (Fig. 2b). However, the output from coverage2cytosine in Bismark skips that site (Fig. 2c), thus indicating that this genomic region has a low level of methylation. On the other hand, the output from DMRfinder includes the novel CpG site (Fig. 2d), revealing that the region actually has a higher methylation level. Furthermore, this particular site is in the middle of a CEBPB binding site (Fig. 2a), as shown by the UCSC genome browser [12]. DNA methylation has been shown to affect the binding of this transcription factor to DNA [13], so this novel CpG site may have a functional consequence in these cells.

The appearance of novel CpG sites is not an insignificantly rare occurrence. For the Lister et al. [9] IMR90 replicate 1a dataset, 53,442 novel CpG sites had methylation information in the alignments that was collected by DMRfinder but ignored by Bismark, such as the example in Fig. 2. Although this amounted to just 0.2% of the nearly 25 million reference CpG sites for which there were methylation counts, these sites have the potential to affect regulatory models in specific cell types or tissues.

Another novel aspect of the first step of DMRfinder is that it allows for the analysis of spatially-linked methylation information. This can be used to determine if methylation differences occur in a nonrandom pattern, which may reflect an alteration in the cellular populations being studied (Additional file 2).

### Clustering CpG sites into regions

Despite the single-base resolution of bisulfite-sequencing data, the inherent noise in such data combined with biological reality indicates that considering sets of spatially adjacent CpG sites as forming a functional unit may be more informative. To this end, the second step of DMRfinder clusters valid CpG sites that are located within a minimum distance parameter of each other. It then modifies these single-linkage clusters by splitting any cluster whose length exceeds a specified threshold into multiple subregions, thus limiting the chaining effect. Since the clustering process is based on the locations of valid CpG sites and not methylation levels, it is not biased in favor of (or opposed to) finding DMRs.

A similar single-linkage clustering algorithm is implemented in the R/Bioconductor package BiSeq [14]. By adjusting the parameters of DMRfinder, clusters were produced that were identical to those from BiSeq (Table 2). However, in analyzing the six IMR90 samples of Lister et al. [9], DMRfinder was more than 31 times faster than BiSeq (Table 3).

**Table 2:** Clusters formed by DMRfinder and BiSeq in analyzing the Lister et al. [9] IMR90 samples

	Number of clusters	Total length (Mbp)	Average length (bp)
DMRfinder (default)	522,963	46.0	88.0
DMRfinder (−r 1 -m 1 -x 1e9 -c 5)	795,637	168.7	212.1
BiSeq	795,637	168.7	212.1

DMRfinder was run using default parameters and an alternative set of parameters matching BiSeq’s clustering approach

**Table 3:** Comparison of run-times (sec) to analyze the Lister et al. [9] IMR90 samples, which had methylation counts at 21-25 million CpG sites

	bsseq	BiSeq	DSS	DMRfinder
Clustering	–	17,367	–	556
Smoothing	18,151	563,738	–	–
Finding DMRs	187	297,501^a^	7,400	552

^a^betaRegression() step only

**Fig. 3:**
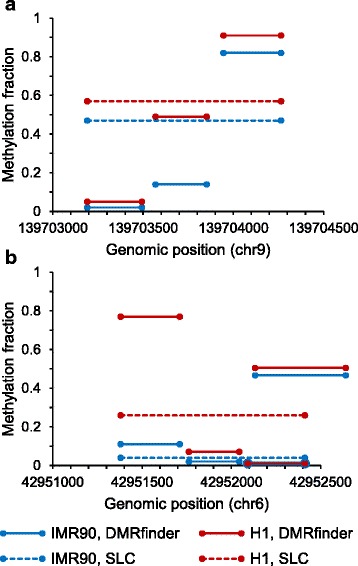
Improved DMR identification by DMRfinder compared to single-linkage clustering (SLC). **a** With SLC (dashed lines), all 40 CpG sites in this region of chromosome 9 were clustered together, and no DMR was called. With DMRfinder (solid lines), the region was split into three subregions, and the middle subregion was determined to be a DMR. **b** With SLC, a DMR was identified as spanning all 37 CpG sites in this region of chromosome 6. After splitting with DMRfinder, only the first subregion was classified as a DMR

In addition, the default modified clustering approach of DMRfinder is superior to pure single-linkage clustering (SLC) in that the former splits large clusters to reduce the chaining effect. This leads to additional DMRs, such as the example shown in Fig. 3a. With SLC, all 40 CpG sites in this region were clustered together, and the subsequent statistical test indicated that there was not a significant methylation difference (q-value 0.12). DMRfinder split this region, and the middle subregion was determined to be a DMR, with a methylation difference of 35% and a q-value of 1.4e-11 (Fig. 3a). The splitting function also helps to define DMRs more precisely, as shown in Fig. 3b. After SLC, this entire region of 37 CpG sites was identified as a DMR (methylation difference 22%, q-value 1.8e-7), but, with splitting, it was determined that only the first subregion accounted for most of the differential methylation (methylation difference 66%, q-value 2.0e-14).

Furthermore, the minimum count parameters of the clustering algorithm in DMRfinder, which have no parallel in BiSeq, ensure that the subsequent statistical tests have sufficient power.

### Testing regions for differential methylation

The final step of the DMRfinder pipeline is to test the regions defined in the previous step for statistically significant methylation differences. The R/Bioconductor package DSS [10] provides the basis for the beta-binomial modeling of methylation counts and subsequent Wald tests. DSS itself calls DMRs by combining CpG sites that individually achieve a high level of statistical significance. By contrast, DMRfinder considers only the clusters, not individual CpG sites. Because methylation counts are conglomerated within each cluster, the number of hypothesis tests is reduced, thereby increasing statistical power.

The performance of DMRfinder was benchmarked against other software packages in two analyses. First, the two IMR90 replicates of Lister et al. [9] were compared against each other, in an analysis that should result in no DMRs (see Additional file 1 for details). The two software packages utilizing data smoothing, bsseq [8] and BiSeq [14], required excessive computational time (Table 3). In addition, bsseq identified 32,643 regions (spanning 18.8Mbp) with methylation differences of at least 10%. BiSeq called 56,398 differentially methylated CpG sites with a *p*-value threshold of 1e-5, but the analysis could not be completed due to time and memory (64Gb) limitations. By contrast, DSS and DMRfinder identified zero and two DMRs, respectively. Though using the same underlying statistics as DSS, DMRfinder required substantially less computational time because it tests regions for differential methylation, not each CpG site (Table 3).

**Fig. 4:**
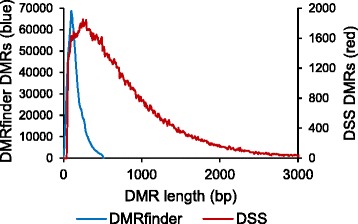
Comparison of DMRs found by DMRfinder and DSS. The clustering approach of DMRfinder leads to DMRs that are more numerous but much shorter than those identified by DSS

The second analysis was to determine DMRs between IMR90 and H1 cells. With DSS, a total of 178,776 regions were identified, covering 141.7 Mbp of the genome. Again using a minimum methylation difference of 10% and a maximum *q*-value of 0.05, DMRfinder called more than six times as many DMRs, though they spanned a similar total genomic distance (172.3Mbp) due to their smaller size (Fig. 4). The overall large quantity of differential methylation found by both DSS and DMRfinder was due to the fact that the H1 cells had a much higher level of CpG methylation than the IMR90 cells (83.6% vs. 67.1%), consistent with previous results [9].

**Fig. 5:**
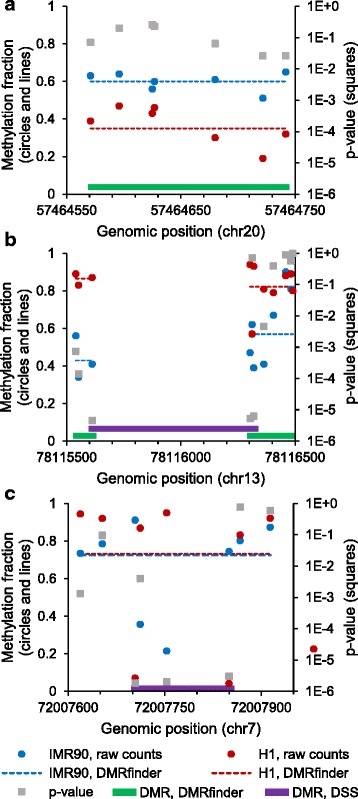
Comparison of DMR identification by DMRfinder and DSS. **a** A region on chromosome 20 that covers seven CpG sites. The methylation levels of IMR90 at all seven sites are higher than those of H1 (circles), but on an individual basis, none of the sites showed significant differential methylation (squares), using the threshold of DSS (*p*-value < 1e-5). With DMRfinder, the sites were clustered together, and the weighted methylation fractions (dashed lines) were determined to be statistically significantly different. **b** DMRs identified by DSS and DMRfinder in a region on chromosome 13. The DMR called by DSS spanned four CpG sites and more than 700 bp (purple box). With DMRfinder, the CpG sites were grouped into two clusters, both of which were classified as DMRs (green boxes). **c** A DMR identified uniquely by DSS on chromosome 7. Two of the CpG sites in the DMR were hypermethylated in IMR90 cells, and two were hypomethylated. For the entire region, DMRfinder calculated a small methylation difference and did not call a DMR

However, most of the DMRs called by DMRfinder (86.9%) had no overlap with those identified by DSS. This was because DMRfinder conglomerates methylation counts within each cluster, thus allowing smaller methylation differences to be detected, whereas DSS requires multiple individual sites to achieve a high level of significance. In fact, 38.5% of the DMRs found by DMRfinder contained CpG sites of which zero were considered significant by DSS, such as the example shown in Fig. 5a. Even though all of the CpG sites in this region had methylation differences of at least 13%, none of them achieved the default DSS statistical threshold of a *p*-value less than 1e-5. On the other hand, DMRfinder classified this region as a DMR, with an overall methylation difference of 25% and a *q*-value of 1.6e-4. This highlights the advantage of testing regions rather than individual CpG sites.

Further examination of the DMRs called by DSS revealed other critical differences between its approach and that of DMRfinder. In order to call a DMR, DSS requires more than half of a set of at least four consecutive CpG sites to have a *p*-value below 1e-5. However, it does not consider the actual genomic positions of the sites, which leads to some DMRs that span large genomic segments (Fig. 4). In the example shown in Fig. 5b, the DMR called by DSS spanned more than 700 bp, with one of the CpG sites far upstream of the others. Nearby CpG sites were excluded from the DMR, because their individual *p*-values failed to meet the threshold. A total of 60.4% of the DMRs identified by DSS had at least one CpG site within 100 bp of one or both ends of the region, like this example (Fig. 5b). By contrast, such DMRs are not called by DMRfinder (except in cases of cluster splitting), because clusters are formed based on genomic distance. In this example, DMRfinder formed two clusters from the CpG sites, each of which was separately classified as a DMR (Fig. 5b).

Another difference between DMRfinder and DSS is that the latter does not consider methylation levels. For example, the DSS-called DMR shown in Fig. 5c had two CpG sites that were hypermethylated in IMR90 cells, and two that were hypomethylated. Thus, although each site had a large methylation difference (more than 50%), the difference in average methylation levels for this DMR was minuscule. Just 0.7% of the DMRs called by DSS contained a mixture of hyper- and hypo-methylated significant sites, like this example. However, this number was diminished because the H1 cell line was simply much more methylated than IMR90, and, in comparisons of samples with similar levels of methylation, this type of DMR would be called more frequently by DSS. By contrast, this type of false positive is not observed with DMRfinder; it tests for differential methylation by region, not by site, and methylation levels are a central part of the analysis. In this example, the calculated methylation difference was less than 1% and no DMR was called by DMRfinder (Fig. 5c).

## Conclusions

DMRfinder identifies differentially methylated regions from MethylC-seq data more efficiently than other software packages. It extracts methylation counts, even from novel CpG sites not found in the reference sequence, and performs a modified single-linkage clustering of CpG sites. This clustering allows for the sensitive and unbiased detection of DMRs via a Bayesian beta-binomial model, leading to reduced false positives and more candidate DMRs than other packages.

Like other MethylC-seq analysis tools, DMRfinder does not allow for more complicated modeling of the data. It identifies individual regions that are differentially methylated but does not consider sets of regions or quantitative traits, and it cannot account for batch effects. These are areas for potential further improvements.

Complete descriptions of the software options of DMRfinder and illustrative examples are found in the UserGuide that accompanies the open-source software on GitHub, and in the provided DMRfinder archive (Additional file 3).

## Availability and requirements

**Project name:** DMRfinder

**Project home page:** github.com/jsh58/DMRfinder

**Operating system:** Linux

**Programming language:** Python, R

**Other requirements:** DSS (www.bioconductor.org/packages/release/bioc/html/DSS.html)

**License:** MIT

**Any restrictions to use by non-academics:** none

## Additional files

Additional file 1: Methods and commands used to analyze the datasets from Lister et al. [9]. (PDF 451 kb)

Additional file 2: Methylation linkage analysis. (PDF 381 kb)

Additional file 3: Archive of DMRfinder, version 0.2. (ZIP 500 kb)

## Abbreviations

DMR: Differentially methylated region
DSS: Dispersion Shrinkage for Sequencing data, an R/Bioconductor package for detecting DMRs
RRBS: Reduced representation bisulfite sequencing
SLC: Single-linkage clustering
