# Supplementary material for: DMRfinder: efficiently identifying differentially methylated regions from MethylC-seq data
Source: BMC Bioinformatics. 2017 Nov 29;18:528. doi: 10.1186/s12859-017-1909-0 (PMC5817627; doi:10.1186/s12859-017-1909-0)
Supplement: Supplementary file 1 — Methods and commands used to analyze the datasets from Lister et al. [9]. (PDF 451 kb) [file 12859_2017_1909_MOESM1_ESM.pdf]

Methods and commands used to analyze the datasets from Lister *et al.* [9]

Datasets from Lister *et al.* [9] were downloaded from the Sequence Read Archive (https://www.ncbi.nlm.nih.gov/sra/). Within each of the eleven groups (Table 1.1), FASTQ files were concatenated, and reads were processed in the following manner. They were first trimmed based on quality scores using qualTrim, available as part of AmpliconTools (github.com/jsh58/AmpliconTools/). Cutadapt [15] was then used to remove the Illumina Universal Adapter sequence and eliminate all reads that were shorter than 21bp. The remaining reads were aligned to the *in silico* bisulfite-converted human genome (hg19) with the Bismark alignment algorithm [6]. Commands are given in Box 1.2.

Table 1.1. Datasets from Lister *et al.* [9].

Dataset	SRA accession	Raw reads (millions)	Aligned reads (millions)
IMR90_r1a	SRX006783	574.0	335.2
IMR90_r1b	SRX006784	640.2	319.3
IMR90_r1c	SRX006785	122.6	90.9
IMR90_r2a	SRX006786	614.0	330.2
IMR90_r2b	SRX006787	697.1	382.6
IMR90_r2c	SRX006788	159.3	117.1
H1_r1a	SRX006789	416.8	252.0
H1_r1b	SRX006782	447.0	333.5
H1_r2a	SRX006239	570.4	363.2
H1_r2b	SRX006240	544.4	306.2
H1_r2c	SRX006241	4.1	2.7

Box 1.2. Commands used to trim and align the IMR90_r1a dataset (other datasets were processed with similar commands).

```
# quality trimming
$ qualTrim  -i imr90_r1a.fastq.gz  -o temp.gz  -l 1  -q 3  -n 21
$ cutadapt  -a AGATCGGAAGAGC  -m 21  -o imr90_r1a_qt.fastq.gz  temp.gz

# alignment to human genome (bismark_genome_preparation executed only once)
$ bismark_genome_preparation  hg19/Sequence/WholeGenomeFasta
$ bismark  hg19/Sequence/WholeGenomeFasta  imr90_r1a_qt.fastq.gz
```

After alignment, methylation count files were generated by the DMRfinder script extract_CpG_data.py. For benchmarking purposes, the same was done using analogous scripts in Bismark (Box 1.3).

Box 1.3. Commands used to compile methylation counts for the IMR90_r1a dataset (other alignment files were processed with similar commands).

```
# DMRfinder
$ samtools view -h imr90_r1a_qt_bismark_bt2.bam | \
  python extract_CpG_data.py  -i -  -o imr90_r1a.cov

# Bismark
$ bismark_methylation_extractor  --single-end  --comprehensive \
    --merge_non_CpG  --bedGraph  imr90_r1a_qt_bismark_bt2.bam
$ coverage2cytosine  --merge_CpG  --output imr90_r1a.c2c \
  --genome_folder hg19/Sequence/WholeGenomeFasta \
  imr90_r1a_qt_bismark_bt2.bismark.cov.gz
```

The methylation count files produced by extract_CpG_data.py were used in two analyses. First, the six IMR90 files were used to determine DMRs between replicates 1 and 2 (replicates 1a/1b/1c vs. 2a/2b/2c; Box 1.4). Since the replicates were artificially divided into three groups, any DMRs thus determined were likely to be false positives.

For the second analysis, the methylation count files within each replicate were combined, leading to four conglomerated count files (IMR90 replicate 1, IMR90 replicate 2, H1 replicate 1, H1 replicate 2). These were used to test for DMRs between IMR90 and H1 cells, via commands similar to those shown in Box 1.4.

In both analyses, DMRfinder labeled any region as significantly differentially methylated if it had a methylation difference of at least 10% and a q-value of at most 0.05.

Language and software versions are given in Table 1.5.

Box 1.4. Commands used to identify DMRs between IMR90 replicate 1 and IMR90 replicate 2.

bsseq

```
$ R
> library(bsseq)
> data <- read.bismark( c('imr90_r1a.cov', 'imr90_r1b.cov',
  + 'imr90_r1c.cov', 'imr90_r2a.cov', 'imr90_r2b.cov', 'imr90_r2c.cov'),
  + c('imr90_r1a', 'imr90_r1b', 'imr90_r1c', 'imr90_r2a', 'imr90_r2b',
  + 'imr90_r2c') )
> data.smooth <- BSmooth(data)
> data.smooth.tstat <- BSmooth.tstat( data.smooth, c('imr90_r1a',
  + 'imr90_r1b', 'imr90_r1c'), c('imr90_r2a', 'imr90_r2b', 'imr90_r2c') )
> data.dmrs <- dmrFinder(data.smooth.tstat)
```

BiSeq

```
$ R
> library(BiSeq)
> df <- data.frame( group=c(rep('imr90_r1', 3), rep('imr90_r2', 3)),
  + row.names=c('imr90_r1a', 'imr90_r1b', 'imr90_r1c', 'imr90_r2a',
  + 'imr90_r2b', 'imr90_r2c') )
> data <- readBismark( c('imr90_r1a.cov', 'imr90_r1b.cov',
  + 'imr90_r1c.cov', 'imr90_r2a.cov', 'imr90_r2b.cov', 'imr90_r2c.cov'),
  + df )
> data.clusters <- clusterSites( data, groups=df$group, perc.samples=1,
  + min.sites=5, max.dist=100 )
> data.meth <- predictMeth(data.clusters)
> data.reg <- betaRegression( formula = ~df$group, link='probit',
  + object=data.meth )
```

DSS

```
$ R
> library(DSS)
> samples <- c('imr90_r1a.cov', 'imr90_r1b.cov', 'imr90_r1c.cov',
  + 'imr90_r2a.cov', 'imr90_r2b.cov', 'imr90_r2c.cov')
> raw <- list()
> for (i in 1:length(samples)) {
  + t <- read.csv( samples[i], sep='\t', header=F )
  + t$V3 <- t$V5 + t$V6
  + t$V4 <- t$V6 <- NULL
  + colnames(t) <- c('chr', 'pos', 'N', 'X')
  + raw[[i]] <- t
  + }
> data <- makeBSseqData( raw, c('imr90_r1a', 'imr90_r1b', 'imr90_r1c',
  + 'imr90_r2a', 'imr90_r2b', 'imr90_r2c')
> dml <- DMLtest( data, group1=c('imr90_r1a', 'imr90_r1b', 'imr90_r1c'),
  + group2=c('imr90_r2a', 'imr90_r2b', 'imr90_r2c') )
> dmr <- callDMR(dml)
```

DMRfinder

```
$ python combine_CpG_sites.py  -o combined.csv  -s 4  imr90_r1a.cov \
  imr90_r1b.cov imr90_r1c.cov imr90_r2a.cov imr90_r2b.cov imr90_r2c.cov
$ Rscript findDMRs.r  -i combined.csv  -o dmrs.csv  -n imr90_r1,imr90_r2
  -q 0.05  imr90_r1a,imr90_r1b,imr90_r1c  imr90_r2a,imr90_r2b,imr90_r2c
```

Table 1.5. Language and software versions used.

Language / Software	Version
DMRfinder	0.2
Python	2.7.12, 3.5.2
R	3.3.1
Bioconductor	3.4
Bismark	0.15.0
Bowtie2	2.2.9
SAMtools	1.3.1
bsseq	1.10.0
BiSeq	1.14.0
DSS	2.14.0

Additional References

[15] Martin M. Cutadapt removes adapter sequences from high-throughput sequencing reads. EMBnet.journal. 2011;17:10-2.
