# Supplementary material for: DMRfinder: efficiently identifying differentially methylated regions from MethylC-seq data
Source: BMC Bioinformatics. 2017 Nov 29;18:528. doi: 10.1186/s12859-017-1909-0 (PMC5817627; doi:10.1186/s12859-017-1909-0)
Supplement: Supplementary file 2 — Methylation linkage analysis. (PDF 381 kb) [file 12859_2017_1909_MOESM2_ESM.pdf]

Methylation linkage analysis

In the analysis of bisulfite-sequencing data, one or more genomic regions may show differential DNA methylation between two conditions, such as a control group and an experimental group. While it is typically assumed that the identified regions reflect a change in the methylation of the original tissue, there remains the possibility that the cellular populations may have shifted between the two groups. For example, the tissue of a pro-inflammatory experimental group may have been infiltrated by a white blood cell population that accounts for the observed methylation differences [16].

Bisulfite sequencing produces single-base resolution methylation data, and, after aligning the reads, programs such as bismark_methylation_extractor conglomerate methylation data by genomic position. The DMRfinder script combine_CpG_sites.py further groups spatially-linked CpG sites into genomic regions. At the end of this analysis, we may find a region that shows differential methylation between a control and experimental group (Box 2.1).

Box 2.1. A sample genomic region, as reported by combine_CpG_sites.py.

```
chrom   start   end   CpG   Control   Experimental
chrZ     102    168    6     0.00         0.40
```

The increased methylation of the experimental group may have resulted from increased DNA methylation due to the experimental treatment, or it may have been caused by an influx of cells with higher methylation levels than the original tissue. From these data values, there is no way to distinguish the two possibilities.

Instead of examining these grouped data, we may wish to consult the single-base resolution results for the six CpG sites found in this genomic region, as produced by DMRfinder's extract_CpG_data.py. As shown in Boxes 2.2 and 2.3, the output file from this program lists the following six values for each CpG site, tab-delimited: chromosome, start and end coordinates, methylation percent, methylated counts, and unmethylated counts.

```
chrZ   102   103    0.0    0    5
chrZ   109   110    0.0    0    5
chrZ   123   124    0.0    0    3
chrZ   140   141    0.0    0    4
chrZ   147   148    0.0    0    5
chrZ   168   169    0.0    0    5
```

Box 2.2. Single-base resolution methylation data for the control sample in the above genomic region.

chrZ	102	103	25.0	1	3
chrZ	109	110	25.0	1	3
chrZ	123	124	50.0	2	2
chrZ	140	141	40.0	2	3
chrZ	147	148	42.9	3	4
chrZ	168	169	50.0	3	3

Box 2.3. Single-base resolution methylation data for the experimental sample in the above genomic region.

These tables are the basis for the summarized results of Box 2.1; the control group had no methylated counts out of twenty-seven, and the experimental group had twelve methylated and eighteen unmethylated (40% methylation). This presentation of the data provides more information on a site-by-site basis, but even so, there are no indications within the data whether or not there may be a shift in cell populations between the two conditions.

However, the original read alignments may provide insight into the pattern of methylation. To examine this pattern within the above region, we can run extract_CpG_data.py while supplying a BED file that designates the above region (Box 2.4).

```
chrZ     100     200     regionA
```

Box 2.4. Designation of a BED region for extract_CpG_data.py.

While collecting methylation data from the alignment file, extract_CpG_data.py will save linked methylation data for any reads that contain at least one CpG site within this BED interval. It will output a summarized version of each read's methylation data, using a simple schema (Box 2.5).

0	unmethylated CpG
1	methylated CpG
-	no data for this site

Box 2.5. Schema for extract_CpG_data.py's linked output. Each read is listed with a string of *n* characters, one for each CpG in the designated region. A read may have no data for a site if it does not cover that site, or if the methylation status cannot be determined because of a sequence variant.

The output of extract_CpG_data.py with the control sample shows seven reads (readC_0 through readC_6) that cover one or more CpG sites within regionA (Box 2.6). Since each column of values represents one CpG site, summing each column individually leads to the summary counts shown in Box 2.2.

```
Region: regionA, chrZ:100-200
Sites: 102, 109, 123, 140, 147, 168
00----    readC_0
00000-    readC_1
000000    readC_2
000000    readC_3
00-000    readC_4
----00    readC_5
-----0    readC_6
```

Box 2.6. Linked methylation information for regionA in the control sample. The six columns for each of the seven reads list the methylation information at the six CpG sites, according to the schema shown in Box 2.5.

Since there are no methylated bases observed in this sample, this set of reads is not particularly informative. However, the experimental sample may show a more interesting pattern of methylation that could take one of several forms.

In scenario one (Box 2.7), the methylated bases appear fairly randomly across the eight reads. Again, note that the methylation counts for each CpG site (the six columns) match the conglomerated counts for the experimental sample shown in Box 2.3.

```
Region: regionA, chrZ:100-200
Sites: 102, 109, 123, 140, 147, 168
00----    readE1_0
10100-    readE1_1
001001    readE1_2
010011    readE1_3
--0110    readE1_4
---100    readE1_5
----00    readE1_6
----11    readE1_7
```

Box 2.7. Linked methylation information for regionA in the experimental sample, scenario one.

It is also possible for the methylation of the experimental sample to have appeared quite differently, as shown in scenario two (Box 2.8).

```
Region: regionA, chrZ:100-200
Sites: 102, 109, 123, 140, 147, 168
00----    readE2_0
00000-    readE2_1
000000    readE2_2
111111    readE2_3
--1111    readE2_4
---000    readE2_5
----00    readE2_6
----11    readE2_7
```

Box 2.8. Linked methylation information for regionA in the experimental sample, scenario two.

The site-by-site methylation profile is identical to that of scenario one, but the methylation has appeared in a distinctly nonrandom fashion. In fact, only three of the eight reads shown any methylation (readE2_3, readE2_4, and readE2_7), and these reads are all entirely methylated. This suggests that the observed methylation differences may be due to the infiltration of an alternative cell type that is fully methylated in this genomic region.

Additional References

[16] Li H, Zheng T, Chen B, Hong G, Zhang W, Shi T, et al. Similar blood-borne DNA methylation alterations in cancer and inflammatory diseases determined by subpopulation shifts in peripheral leukocytes. Br J Cancer. 2014;111:525-31.
