# Supplementary material for: DMRfinder: efficiently identifying differentially methylated regions from MethylC-seq data
Source: BMC Bioinformatics. 2017 Nov 29;18:528. doi: 10.1186/s12859-017-1909-0 (PMC5817627; doi:10.1186/s12859-017-1909-0)
Supplement: Supplementary file 3 — Archive of DMRfinder, version 0.2. (ZIP 500 kb) [file 12859_2017_1909_MOESM3_ESM.zip › DMRfinder/UserGuide.pdf]

DMRfinder: Identifying differentially methylated regions from MethylC-seq data

John M. Gaspar

June 13, 2017

Contents

1 Introduction 1
1.1 Quick start 2
1.2 Software prerequisites 2
2 Alignment 3
3 Extracting methylation counts 4
4 Clustering CpG sites into regions 6
5 Testing regions for differential methylation 9
6 Miscellaneous 13
6.1 Universal options 13
6.2 References 13
6.3 Contact 13
Appendix A. Illustrative examples with combine_CpG_sites.py. 14
Appendix B. Illustrative examples with findDMRs.r. 16

1 Introduction

DMRfinder efficiently identifies genomic regions with differentially methylated CpG sites from high-throughput MethylC-seq datasets (sequencing preceded by bisulfite treatment). Following alignment with Bismark [1], DMRfinder extracts methylation counts and clusters the CpG sites into genomic regions. It then performs pairwise comparisons to identify differentially methylated regions (DMRs) using the statistical framework of the Bioconductor package DSS [2].

This document describes the usage and parameters of DMRfinder.

1.1 Quick start

Given:
- '/path/to/genome' -- a file path to a reference genome
- C1.fastq, C2.fastq: single-end sequence files for two samples in the group 'Control'
- E1.fastq, E2.fastq: single-end sequence files for two samples in the group 'Exptl'

Looking for:
- Genomic regions that are differentially methylated between 'Control' and 'Exptl'

Step 1: Alignment
```
$ bismark_genome_preparation  /path/to/genome
$ bismark  /path/to/genome  C1.fastq
$ bismark  /path/to/genome  C2.fastq
$ bismark  /path/to/genome  E1.fastq
$ bismark  /path/to/genome  E2.fastq
```

Step 2: Extract methylated/unmethylated counts from alignment files
```
$ samtools view -h C1_bismark_bt2.bam | \
  python extract_CpG_data.py  -i -  -o C1.cov
$ samtools view -h C2_bismark_bt2.bam | \
  python extract_CpG_data.py  -i -  -o C2.cov
$ samtools view -h E1_bismark_bt2.bam | \
  python extract_CpG_data.py  -i -  -o E1.cov
$ samtools view -h E2_bismark_bt2.bam | \
  python extract_CpG_data.py  -i -  -o E2.cov
```

Step 3: Cluster CpG sites into regions
```
$ python combine_CpG_sites.py  -o combined.csv C1.cov C2.cov E1.cov E2.cov
```

Step 4: Test regions for differential methylation
```
$ Rscript findDMRs.r  -i combined.csv  -o results.csv  \
    -n Control,Exptl  C1,C2  E1,E2
```

1.2 Software prerequisites

- DMRfinder (github.com/jsh58/DMRfinder)
- Bismark (www.bioinformatics.babraham.ac.uk/projects/bismark/)
- Bowtie2 (bowtie-bio.sourceforge.net/bowtie2/index.shtml)
- DSS (www.bioconductor.org/packages/release/bioc/html/DSS.html). This package requires R (www.r-project.org/) and Bioconductor (www.bioconductor.org/install/). DMRfinder scripts have been tested with Rscript version 3.3.1.
- Python (www.python.org/downloads/). DMRfinder scripts have been tested with Python versions 2.7.12 and 3.5.2.

Optional:
- SAMtools (samtools.sourceforge.net). If this is installed, the output from Bismark will be converted to BAM; otherwise, the output will be gzip-compressed. Either output can be analyzed by DMRfinder, as described in Section 3 below.

2 Alignment

To identify the methylation status of genomic cytosines, the high-throughput sequence reads from a MethylC-seq run first must be aligned to a reference genome. To accomplish this, Bismark [1] performs an *in silico* bisulfite conversion of both the reads and the reference genome, and then uses Bowtie2 [3] to align the reads to the reference. Reads with a single optimal alignment are further analyzed for methylation status; reference cytosines that were sequenced as thymines are labeled unmethylated, and those that remained as cytosines are labeled methylated. All methylation calls are made with respect to the forward strand, so for reads that align to the reverse strand of the genome, methylation calls appear on the guanine bases. Bismark classifies each methylation call based on the sequence context (CpG/CHG/CHH).

There are many command-line options in Bismark, which are described in the User Guide (rawgit.com/FelixKrueger/Bismark/master/Docs/Bismark_User_Guide.html). Of note, because Bismark relies on the alignments produced by Bowtie2, most of the Bowtie2 command-line options are available with Bismark as well (a full description of the Bowtie2 options can be found at bowtie-bio.sourceforge.net/bowtie2/manual.shtml).

Following alignment, some researchers choose to remove reads that may be PCR duplicates. We do not recommend using Bismark's deduplication script for this purpose; it simply keeps the first read at a given position in the alignment file and eliminates the rest, regardless of the reads' sequences or methylation information.

A bug related to ambiguous read alignments crept into version 0.16.0 of Bismark and remains in the most recent version (0.18.1, as of June 2017). Until this is fixed, we recommend version 0.15.0 (github.com/FelixKrueger/Bismark/archive/v0.15.0.tar.gz).

3 Extracting methylation counts

The DMRfinder script extract_CpG_data.py converts the output from Bismark's aligner into a table of methylated/unmethylated counts at each CpG site. The table is sorted by chromosome (based on the order of reference sequence names in the header of the SAM) and position, which is the 1-based coordinate of the cytosine of the CpG. The script also merges methylation counts, in that it sums counts for both the cytosine and the guanine of each CpG site (this occurs when reads align to different strands of the reference -- see Section 2 above).

```
Usage: python extract_CpG_data.py  [options]  -i <input>  -o <output>
    -i <input>    SAM alignment file produced by Bismark (must have
                    a header, 'XM' methylation strings, and 'XG'
                    genome designations; can use '-' for stdin)
    -o <output>   Output file listing counts of methylated and
                    unmethylated CpGs, merged and sorted
  Options:
    -m <int>      Minimum coverage (methylation counts) to report a
                    CpG site (def. 1)
    -s            Report strand in third column of output
    -b <file>     BED file listing regions for which to collect
                    linked methylation data
```

```
    -i <input>    SAM alignment file produced by Bismark (must have
                    a header, 'XM' methylation strings, and 'XG'
                    genome designations; can use '-' for stdin)
```

A SAM alignment file produced by Bismark, whether gzip-compressed or not, can be used directly as the <input> file. However, a BAM file must be converted to SAM, or it can be piped in via SAMtools, e.g.:

```
$ samtools view -h <BAM> | python extract_CpG_data.py  -i -  -o <output>
```

```
    -o <output>   Output file listing counts of methylated and
                    unmethylated CpGs, merged and sorted
```

Each line of the output lists the following six values for a single CpG, tab-delimited:

<chrom>	chromosome name
<chromStart>	1-based position of the cytosine in the CpG
<chromEnd>	<chromStart> + 1
<percent>	percent methylation at this site
<methylated>	count of methylated cytosines
<unmethylated>	count of unmethylated cytosines

```
-m <int>      Minimum coverage (methylation counts) to report a
                CpG site (def. 1)
```

Any sites that do not have at least the specified number of methylation counts will be excluded from the output. By default, all sites with at least one count are reported.

```
-s            Report strand in third column of output
```

With this option, the 3rd column of the output, instead of <chromEnd>, will contain the strand. Because this is a merged output, the strand will invariably be '+'.

```
-b <file>     BED file listing regions for which to collect
                linked methylation data
```

On separate lines, the input BED file should list, for each genomic region of interest, chromosome name, 1-based start and end coordinates, and unique region name, tab-delimited. For example:

```
chrZ   100   200   regionA
```

The output file, <file>_linked.txt, will list, for each BED region, the methylation data for reads that cover at least one CpG in the region. The methylation data is summarized using '0' for an unmethylated CpG, '1' for methylated, and '-' for no data. For example:

```
Region: regionA, chrZ:100-200
Sites: 102, 109, 123, 140, 147, 168
00100-  read1
001100  read2
--0000  read3
```

This shows that, in regionA, there were six CpG sites, corresponding to the six columns of data for the three reads. The first read ('read1') had unmethylated CpGs at positions 102, 109, 140, and 147, while the CpG at position 123 was methylated. The last CpG, at position 168, was not covered by this read. The methylation data for the six CpG sites are similarly shown for read2 and read3.

Note: this script is specific for producing merged CpG methylation counts. To get comprehensive methylation information, such as CHG/CHH methylation counts or M-bias plots, one can use the Bismark script bismark_methylation_extractor. To produce a merged output similar to that of extract_CpG_data.py, one must also run the Bismark script coverage2cytosine on the bismark_methylation_extractor output.

4 Clustering CpG sites into regions

The DMRfinder script combine_CpG_sites.py takes one or more samples' methylation counts at individual CpG sites and produces a single output. It clusters adjacent CpG sites into genomic regions, following a three-step procedure as described below, and sums the methylation data within the regions for each sample.

```
Usage: python combine_CpG_sites.py  [options]  -o <output>  [<input>]+
    [<input>]+    One or more files, each listing methylation counts
                    for a particular sample
    -o <output>   Output file listing genomic regions and combined
                    methylation counts for each sample
  Options:
    To consider a particular CpG:
      -r <int>    Min. number of counts at a position (def. 3)
      -s <int>    Min. number of samples with -r counts (def. 1)
    To analyze a region of CpGs:
      -d <int>    Max. distance between CpG sites (def. 100)
      -c <int>    Min. number of CpGs in a region (def. 3)
      -x <int>    Max. length of a region (def. 500)
    To report a particular result:
      -m <int>    Min. total counts in a region (def. 20)
    Other:
      -f          Report methylation fraction for each sample
```

```
    [<input>]+    One or more files, each listing methylation counts
                    for a particular sample
```

These headerless files must have six tab-delimited fields per line, with the first, second, fifth, and sixth columns being chromosome, position, methylated counts, and unmethylated counts, respectively (the third and fourth columns are not used). This is the format produced by extract_CpG_data.py, as well as coverage2cytosine in Bismark.

```
    -o <output>   Output file listing genomic regions and combined
                    methylation counts for each sample
```

The header line of this tab-delimited output file lists the following: chr, start, end, CpG, and two columns for each input file (the sample name [the basename of the input file, minus the suffix] followed by '-N', and the sample name followed by '-X'). In the following lines, the first four columns give the location of a genomic region ('start' and 'end' are the 1-based positions of the cytosines in the first and last CpG sites of the region) and the number of CpG sites it contains. The remaining columns give the methylation data for each sample; the '-N' column gives the total counts for the sample in the region, and the '-X' column gives the methylated counts.

The parameters controlling the three-step process of combining CpG sites into regions are described below. Illustrative examples are provided in Appendix A.

Step one is to determine the list of valid CpG sites that meet the coverage criteria defined by the two parameters -r and -s.

```
To consider a particular CpG:
  -r <int>    Min. number of counts at a position (def. 3)
  -s <int>    Min. number of samples with -r counts (def. 1)
```

In a given sample, any CpG sites that do not have sufficient counts to meet the -r threshold will be disregarded. Note that both methylated and unmethylated counts are considered; the methylation fraction is not taken into account. The default value is 3. Setting '-r 1' means that all sites will be analyzed.

Once all the samples' individual CpG sites are determined, the sites without sufficient counts in the number of samples set by -s are then invalidated. The default value of 1 means that only one sample need meet the threshold, so no sites will be invalidated.

Step two is to perform a modified single-linkage clustering of the CpG sites into genomic regions, as established by the three parameters -d, -c, and -x.

```
To analyze a region of CpGs:
  -d <int>    Max. distance between CpG sites (def. 100)
  -c <int>    Min. number of CpGs in a region (def. 3)
  -x <int>    Max. length of a region (def. 500)
```

Any CpG sites that are within the distance set by -d are placed into the same cluster. For example, with the default value of 100bp, CpGs at positions 100, 120, 200, and 300 will form one cluster. Another CpG at position 401 will not join the cluster, because it is more than 100bp from the nearest CpG site.

To be reported in the output file, a cluster must have at least the number of CpG sites defined by the -c parameter. The default value is 3. Setting '-c 1' means that all regions will be reported.

The -x parameter sets the (ideal) maximum length for a region (default 500bp). This option effectively limits the chaining effect of the single-linkage clustering. However, this parameter is not strictly enforced; a region longer than the specified length will be split only in such a way that the subclusters meet the -c threshold. If such a split is not possible, the original region will be kept as is. Note that, following a split, each subcluster must individually meet the -m threshold as described below.

Step three is to perform a final filtering based on the number of counts in each sample individually, using the -m parameter.

```
    To report a particular result:
      -m <int>    Min. total counts in a region (def. 20)
```

For each region, the sum of methylated and unmethylated counts at valid CpG sites is calculated per sample. Any sample for which this sum does not meet the -m threshold is reported as 'NA' for both '-N' and '-X' columns in the output file; otherwise, the total sum is reported under '-N' and the methylated sum under '-X'. Note that a valid CpG site is considered valid for all samples, even those that failed to meet the -r threshold.

```
    Other:
      -f          Report methylation fraction for each sample
```

With this option, the output file will contain only one column per sample, which lists the methylation fraction in each region. Note that this is calculated as the sum of methylated counts at the CpG sites within a region divided by the total sum of counts, not the average of the methylation fractions at those CpG sites.

Illustrative examples of the usage of combine_CpG_sites.py are provided in Appendix A.

5 Testing regions for differential methylation

The DMRfinder script findDMRs.r conducts pairwise tests of sample groups to find genomic regions that are differentially methylated. The underlying statistics are based on the beta-binomial hierarchical modeling and Wald test implemented in the Bioconductor package DSS [2].

```
Usage: Rscript findDMRs.r  [options]  -i <input>  -o <output>  \
             <groupList1>  <groupList2>  [...]
    -i <input>    File listing genomic regions and methylation counts
    -o <output>   Output file listing methylation results
    <groupList>   Comma-separated list of sample names (at least two
                    such lists must be provided)
  Options:
    -n <str>      Comma-separated list of group names
    -k <str>      Column names of <input> to copy to <output> (comma-
                    separated; def. "chr, start, end, CpG")
    -s <str>      Column names of DSS output to include in <output>
                    (comma-separated; def. "mu, diff, pval")
    -c <int>      Min. number of CpGs in a region (def. 3)
    -d <float>    Min. methylation difference between sample groups
                    ([0-1]; def. 0.10)
    -p <float>    Max. p-value ([0-1]; def. 0.05)
    -q <float>    Max. q-value ([0-1]; def. 1)
    -up           Report only regions hypermethylated in later group
    -down         Report only regions hypomethylated in later group
    -t <int>      Report regions with at least <int> comparisons
                    that are significant (def. 1)
```

```
    -i <input>    File listing genomic regions and methylation counts
```

This tab-delimited file must have columns labeled chr, start, end, and CpG. In addition, for each of the samples to be analyzed, there must be two columns, the sample name followed by '-N' and the sample name followed by '-X'. This is the format produced by combine_CpG_sites.py.

```
    -o <output>   Output file listing methylation results
```

This tab-delimited output file lists any regions judged as differentially methylated, based on the specified thresholds (-c/-d/-p/-q/-up/-down/-t, see below). For each region, the same four columns (chr, start, end, CpG) will be reported, plus statistical results from DSS (methylation levels, differences, and p-values; see -k, -s below).

```
<groupList>   Comma-separated list of sample names (at least two
                such lists must be provided)
```

These are the lists of samples for each group between which methylation differences are to be ascertained. The sample names must match those found in the header of the input file (not including the '-N' and '-X'). As indicated, at least two such lists must be provided. If more than two groups are provided, this script will conduct pairwise tests for each two-group combination.

An individual sample cannot be included multiple times, not even in different groups. Not every sample in the input file need be analyzed.

```
-n <str>      Comma-separated list of group names
```

This list should correspond to the <groupList> order on the command-line. The names are how each group will be referred to in the output file. By default, if this option is not supplied, group names are constructed from the list of sample names, joined by '_'.

```
-k <str>      Column names of <input> to copy to <output> (comma-
                separated; def. "chr, start, end, CpG")
```

These are the fields from the input file that are included in the output file. The default values are the four columns that are required in the input; columns selected with -k will be appended to this list. For example, if an input file contains genomic annotations, specifying the column header(s) of the annotations with -k will ensure that these fields are copied to the output file.

```
-s <str>      Column names of DSS output to include in <output>
                (comma-separated; def. "mu, diff, pval")
```

These are the fields of the DSS output that are included in the output file. The default values are mu (methylation fraction), diff (methylation difference), and pval (p-value); columns selected with -s will be appended to this list. Options available with DSS (2.14.0) are the following: phi, diff.se, stat, fdr. When -q is specified (see below), 'fdr' is automatically selected.

The methylation fraction is reported for each group individually, as 'group:mu'. For each group-group comparison, slightly different values for mu may be reported for a

given group; these values are averaged for the output file. The same is true for other values that are reported for each group (e.g. ‘phi’).

The methylation difference and p-value are reported for each group-group comparison, as ‘group1->group2:diff’ and ‘group1->group2:pval’. Differences are given as group2 with respect to group1, so values below zero represent hypomethylation in group2. Because of occasional inconsistencies in reported methylation levels (as described in the previous paragraph), a given ‘group1->group2:diff’ might not exactly equal ‘group2:mu’ minus ‘group1:mu’.

```
-c <int>      Min. number of CpGs in a region (def. 3)
```

Any regions not containing at least the specified number of CpG sites will be eliminated. This parameter is equivalent to -c in combine_CpG_sites.py.

```
-d <float>    Min. methylation difference between sample groups
                ([0-1]; def. 0.10)
```

This is the minimum methylation difference that is considered significant. The default value of 0.10 means that there must be at least a 10% methylation difference, plus or minus, between two groups. Specifying ‘-d 0’ means that no regions will be rejected on this basis, except for those listed as ‘NA’.

```
-p <float>    Max. p-value ([0-1]; def. 0.05)
```

This is the maximum p-value that is considered significant. The default value of 0.05 means that a group-group comparison must have a p-value at or below this threshold. Specifying ‘-p 1’ means that no regions will be rejected on this basis, except for those listed as ‘NA’.

```
-q <float>    Max. q-value ([0-1]; def. 1)
```

This is the maximum q-value (false discovery rate) that is considered significant. The default value of 1 means that no regions will be rejected on this basis. When this option is used, ‘fdr’ is automatically added as an output column for each group-group comparison.

```
-up            Report only regions hypermethylated in later group
-down          Report only regions hypomethylated in later group
```

These options limit which regions are reported. The order of groups given on the command-line determines which group is considered 'later'.

```
-t <int>       Report regions with at least <int> comparisons
                 that are significant (def. 1)
```

This parameter determines how many group-group comparisons in a region must meet the -d/-p/-q/-up/-down threshold(s) to be reported. The default value of 1 means that only one comparison must be considered significant to report the region; even if other comparisons do not meet the threshold(s), their results for the region will be included as well.

Specifying '-t 0' means that the -d/-p/-q/-up/-down parameters will be ignored. All regions will be reported, regardless of the DSS results (including 'NA'). To keep all results except for the 'NA', one can specify '-t 1 -c 1 -d 0 -p 1'.

Illustrative examples of the usage of findDMRs.r are provided in Appendix B.

6 Miscellaneous

6.1 Universal options

The following options work for all scripts in DMRfinder:

```
-h/--help     Print the usage message
--version     Print the version and copyright
-v            Run the script in verbose mode (printing various
                updates and statistics to stdout/stderr)
```

6.2 References

[1] Krueger F, Andrews SR. Bismark: a flexible aligner and methylation caller for Bisulfite-Seq applications. Bioinformatics. 2011 Jun 1;27(11):1571-2.

[2] Feng H, Conneely KN, Wu H. A Bayesian hierarchical model to detect differentially methylated loci from single nucleotide resolution sequencing data. Nucleic Acids Res. 2014 Apr;42(8):e69.

[3] Langmead B, Salzberg SL. Fast gapped-read alignment with Bowtie 2. Nat Methods. 2012 Mar 4;9(4):357-9.

6.3 Contact

DMRfinder
Copyright © 2016 John M. Gaspar (jsh58@wildcats.unh.edu)

The software was created at Rutgers University, under the support of NIH grant 1R01AT009152.

Appendix A. Illustrative examples with combine_CpG_sites.py.

In this section, we will demonstrate the three-step process for producing genomic regions as implemented in combine_CpG_sites.py. We will use a simplified set of methylation counts for two samples, C1 and C2 (Box A1).

```
      C1.cov                          C2.cov
chrZ   100   0   1            chrZ   100   1   2
chrZ   120   1   2            chrZ   120   2   3
chrZ   200   0   4            chrZ   200   1   5
chrZ   300   3   3            chrZ   300   0   2
chrZ   401   2   6            chrZ   401   3   3
chrZ   450   3   5            chrZ   450   4   5
chrZ   600   5   2            chrZ   600   6   3
chrZ   625   4   2            chrZ   625   8   0
chrZ   650   5   1            chrZ   650   7   1
chrZ   700   3   2            chrZ   700   3   4
```

Box A1. Methylation counts for samples C1 and C2, as produced by extract_CpG_data.py. For simplification, the third and fourth columns are not shown.

These files were analyzed using the following command.

```
$ python combine_CpG_sites.py  -o results.csv  C1.cov  C2.cov
```

With the default parameters (-r 3 -s 1 -d 100 -c 3 -x 500 -m 20), combine_CpG_sites.py utilized the following procedure.

First, with the default of -r 3, it determined which CpG sites in each sample had at least three methylated/unmethylated counts (the sum of the last two columns of the data). For sample C1, all the sites met this criterion except for position 100; in sample C2, only position 300 failed to have at least three counts. After this, the collective validity of the CpG sites was evaluated; with the default of -s 1, only one sample needed to meet the -r threshold at each location. Therefore, all CpG sites shown in Box A1 were considered valid. Note that, if '-s 2' had been set, the CpG sites at positions 100 and 300 would have been discarded, because fewer than two samples had sufficient counts at those sites.

Next, regions of adjacent CpG sites were defined. With the default maximum distance between CpG sites of 100bp (-d 100), this dataset was clustered into three groups: (100, 120, 200, 300); (401, 450); (600, 625, 650, 700). After this, any clusters smaller than the minimum size were eliminated; with the default value of three (-c 3), the second cluster was removed, leaving (100, 120, 200, 300) and (600, 625, 650, 700). The maximum length parameter (-x 500) did not affect the remaining clusters, since neither was longer than 500bp.

Finally, the methylation counts within these clusters were summed for each sample, and those failing to meet the threshold established by the -m parameter were reported as 'NA'. With the first cluster, the sum of counts for sample C1 was 14, and that for sample C2 was 16. Therefore, since neither sample met the threshold (-m 20), this cluster was not even reported in the output. For the remaining cluster, the sum of methylated counts for both samples was placed in their respective '-X' columns, and the total sum of counts was in the '-N' columns. The resulting table is shown in Box A2.

Box A2. Output from combine_CpG_sites.py, default parameters.

chr	start	end	CpG	C1-N	C1-X	C2-N	C2-X
chrZ	600	700	4	24	17	32	24

Other results produced by combine_CpG_sites.py using parameters other than the defaults are shown below.

Box A3. Output from combine_CpG_sites.py, -m 15.

chr	start	end	CpG	C1-N	C1-X	C2-N	C2-X
chrZ	100	300	4	NA	NA	16	4
chrZ	600	700	4	24	17	32	24

Box A4. Output from combine_CpG_sites.py, -m 10.

chr	start	end	CpG	C1-N	C1-X	C2-N	C2-X
chrZ	120	300	4	14	4	16	4
chrZ	600	700	4	24	17	32	24

Box A5. Output from combine_CpG_sites.py, -r 2 -s 2 -m 10.

chr	start	end	CpG	C1-N	C1-X	C2-N	C2-X
chrZ	120	300	3	13	4	13	3
chrZ	600	700	4	24	17	32	24

Box A6. Output from combine_CpG_sites.py, -c 2 -x 50 -m 10.

chr	start	end	CpG	C1-N	C1-X	C2-N	C2-X
chrZ	200	300	2	10	3	NA	NA
chrZ	401	450	2	16	5	15	7
chrZ	600	625	2	13	9	17	14
chrZ	650	700	2	11	8	15	10

Appendix B. Illustrative examples with findDMRs.r.

In this section, we will outline how the statistical results from DSS are incorporated into the output of this script. We will use a simplified dataset for two groups (Control and Experimental), each of which had two samples (C1/C2 and E1/E2). The methylation counts, as produced by combine_CpG_sites.py, are shown in Box B1.

Box B1. Input to findDMRs.r.

```
chr   start   end   CpG   C1-N   C1-X   C2-N   C2-X   E1-N   E1-X   E2-N   E2-X
chrZ  100     300   4     14      4     16      4     21     10     30      8
chrZ  600     700   4     24     17     32     24     28     15     35     16
```

This file was analyzed using the following command, resulting in the output shown in Box B2.

```
$ Rscript findDMRs.r -i input.csv -o out.csv C1,C2 E1,E2 -n Ctrl,Exp
```

Box B2. Output from findDMRs.r, default parameters.

```
chr   start end CpG   Ctrl:mu   Exp:mu  Ctrl->Exp:diff Ctrl->Exp:pval
chrZ   600  700  4   0.727412  0.493122   -0.2342893     0.0302165
```

This shows that, for the genomic region of chrZ:600-700, the methylation levels (‘mu’) in the Control and Experimental groups were 72.7% and 49.3%, respectively. Therefore, the difference of -23.4% easily met the 10% default threshold, and the p-value of 0.03 was below the default value of 0.05. The other region, chrZ:100-300, was not reported in the output because it failed to meet one or both thresholds.

To get the mu, diff, and pval results for every region (except those whose results are just ‘NA’), we can run findDMRs.r with ‘-d 0 -p 1’ (Box B2).

Box B2. Output from findDMRs.r, -d 0 -p 1.

```
chr   start end CpG   Ctrl:mu   Exp:mu  Ctrl->Exp:diff Ctrl->Exp:pval
chrZ   100  300  4   0.273988  0.362521    0.0885337     0.4701449
chrZ   600  700  4   0.727412  0.493122   -0.2342893     0.0302165
```

From this we can see that the first genomic region (chrZ:100-300) had a methylation difference less than the 10% threshold, and a large p-value as well.
